# The Skin

**Published:** 1890-06

**Authors:** 


### THE SKIN.

A normal condition of the skin is the chief protection against a cold. Three-fourths of the sufferers from catarrhal pneumonia or chronic bronchitis are found to be in the habit of neglecting the skin. Their skin has become degraded, and is no longer a protective covering for the body.

The skin needs to be hardened by the use of the flesh brush, the cold douche, the air bath and by frequent change of underclothing. Active exercise needs to be added, to keep the tissues from clogging. The time to cure the patient is before he gets the cold.

